# Bradycardia and coronavirus disease 2019: What is behind?

**DOI:** 10.1002/clc.23701

**Published:** 2021-07-30

**Authors:** Fotios Barkas, Aris Bechlioulis, Evangelos Liberopoulos

**Affiliations:** ^1^ Department of Internal Medicine University Hospital of Ioannina Ioannina Greece; ^2^ Department of Cardiology University Hospital of Ioannina Ioannina Greece

To the Editor,

We read with interest the paper by Kumar et al. on the prevalence of bradycardia and its association with mortality in patients with coronavirus disease 2019 (COVID‐19).^1^ Among 1053 patients hospitalized with COVID‐19, 24.9% had absolute (<60 bpm) and 13.0% profound (<50 bpm) bradycardia.^1^ Subjects with absolute bradycardia exhibited a higher mortality risk compared with those with normal heart rhythm (odds ratio: 6.59; 95% confidence interval: 2.83–15.36).^1^

It is well established that SARS‐CoV‐2 infection is associated with cardiovascular complications, such as myocardial infarction, myocarditis, and rhythm abnormalities, including bradycardia.^2^, ^3^ Severe hypoxia, inflammatory damage of cardiac pacemaker cells in the setting of myocarditis, myocardial ischemia, electrolyte and intravascular volume imbalances, along with cytokine release syndrome and drug‐associated side effects have been proposed as probable causes of arrhythmias in this setting.^3^

Corticosteroids, remdesivir, tocilizumab, and anakinra are the commonly used drugs in hospitalized patients with COVID‐19.^4^, ^5^ Among them, remdesivir has been previously associated with bradycardia.^6^, ^7^ Mitochondrial dysfunction prompted by the strong affinity of remdesivir for human mitochondrial RNA polymerase (h‐mtRNAP), along with atrioventricular nodal inhibition due to its resemblance with adenosine, could be potential mechanisms.^7^ Kumar et al. reported that 28.7% of patients treated with remdesivir developed an absolute bradycardic response.^1^ In this regard, a formal statistical comparison between patients on versus off remdesivir would be useful in determining whether this medication was associated with increased bradycardia risk in their study. Moreover, the association of day of remdesivir administration with bradycardia diagnosis should be explored. Finally, it would be quite interesting if the authors investigated any association between remdesivir‐associated bradycardia and mortality.

Bradycardia is frequent and clinically relevant in patients with COVID‐19. Additional studies are needed to elucidate its underlying pathophysiological mechanisms.

## CONFLICT OF INTEREST

The authors have no conflicts of interest to declare.
